# Long-Term Impact of Preterm Birth on Exercise Capacity in Healthy Young Men: A National Population-Based Cohort Study

**DOI:** 10.1371/journal.pone.0080869

**Published:** 2013-12-06

**Authors:** Jenny Svedenkrans, Ewa Henckel, Jan Kowalski, Mikael Norman, Kajsa Bohlin

**Affiliations:** 1 Department of Clinical Science, Intervention and Technology, Karolinska Institutet, Stockholm, Sweden; 2 Department of Neonatology, Karolinska University Hospital Huddinge, Stockholm, Sweden; Institute of Psychology, Chinese Academy of Sciences, China

## Abstract

**Background:**

Increasing numbers of survivors of preterm birth are growing into adulthood today. Long-term health-effects of prematurity are still poorly understood, but include increased risk for diabetes, obesity and cardiovascular diseases in adult life. To test if reduced physical fitness may be a link in the causal chain of preterm birth and diseases in later life, the association of preterm birth and adult exercise capacity was investigated. The hypothesis was that preterm birth contributes independently of other risk factors to lower physical fitness in adulthood.

**Methods and Findings:**

Population-based national cohort study of all males conscripting for military service in 1993–2001 and born in Sweden 1973–1983, n = 218,820. Data were retrieved from the Swedish Conscript Register, the Medical Birth Register and the Population and Housing Census 1990. Primary outcome was the results from maximal exercise test (W_max_ in Watt) performed at conscription. Association to perinatal and socioeconomic risk factors, other co-variates and confounders were analysed. General linear modelling showed that preterm birth predicted low W_max_ in a dose-response related pattern, with 25 Watt reduction in W_max_ for the lowest gestational ages, those born ≤27 weeks. Low birth weight for gestational age also independently predicted low W_max_ compared to normal and high birth weight (32 Watt reduction for those with a birth weight Standard Deviation Score <2). Low parental education was significantly associated with reduced W_max_ (range 17 Watt), as well as both low and high current BMI, with severe obesity resulting in a 16 Watt deficit compared to W_max_ top performance.

**Conclusion:**

Being born preterm as well as being born small for gestational age predicts low exercise capacity in otherwise healthy young men. The effect size of being born preterm equal or exceed that of other known risk factors for unfitness in adults, such as low parental education and overweight.

## Introduction

Advances in perinatal medicine have dramatically increased survival after preterm birth [1], [2]. Although this progress is very welcome for women delivering preterm and their families, there is an increasing concern that preterm birth may be an emerging risk factor for chronic lung problems [3]–[5], arterial hypertension [6]–[8] and type 2 diabetes [9]–[11], which predicts accelerated aging, cardiovascular disease and early death [12].

Physical Activity (PA) is important for well-being and has a well-documented favourable effect on many of the established risk factors for cardiovascular disease [13]–[15], such as diabetes [15], [16], hypertension [17] and overweight [15], [18]. Most studies report PA as health promoting independently of other factors [13], but the effect may also be mediated through changes in insulin sensitivity [15], physical fitness and body weight [15], [18].

Observational studies indicate that subjects born very or extremely preterm end up less physically active or less resilient to PA in later life [5], [19]–[22], however contemporary and large population-based follow-up studies on PA and exercise capacity in all adult survivors of preterm birth are lacking. Available reports are small [3]–[5], [19], [23], [24], some are old [24], and commonly focus on lung function in childhood [3], [4], [23]–[25] as bronchopulmonary dysplasia is a major morbidity of prematurity that may affect long-term respiratory health and influence exercise capacity. Results are conflicting, some showing decreased [3], [5], [25] and others normal exercise performance at follow-up [24], [26], [27]. Furthermore, several previous studies have used birth weight as a proxy for GA or included subjects according to birth weight [3], [5], [19], [23], resulting in a selection bias towards growth restricted infants. Any contribution to later PA and exercise capacity from fetal growth restriction - which is over-represented in pregnancies ending preterm - remains to be clarified.

We hypothesized that preterm birth is an independent risk factor for reduced exercise capacity in young adults. To test this hypothesis and control for potential confounding from fetal growth restriction, socioeconomic and familial factors, we studied the results of a graded cycling exercise test performed within the assessment for military service in a large, population-based cohort of 18-year-old Swedish men born 1973 through 1983.

## Methods

### Study Design

This cohort study was based on data from four population-based Swedish registers: the Medical Birth Register (MBR), the Conscript Register, the Population and Housing Census 1990 and the Multigeneration Register. The national registration number, assigned to each Swedish resident at birth, was used for individual record linkage. Linking of data from the different registers was performed by the Central Bureau of Statistics Sweden. The final dataset that was released to the authors and used for analysis was anonymous. Individual consent was not obtained from subjects included in the study and the approving ethical review board waived consent.

The MBR contains data on >99% of all births in Sweden. Starting at the first prenatal visit, information is prospectively collected on standardized forms and forwarded to the register. The MBR has been validated, and the quality was considered high [28].

**Figure 1:**
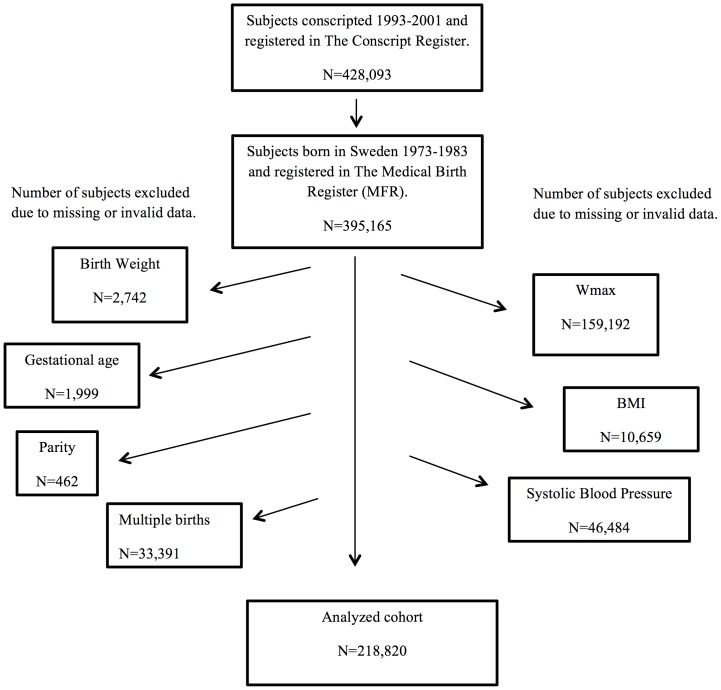
Formation of analyzed cohort. One subject may have missing data in more than one category. W_max_ = maximal exercise capacity, BMI = Body Mass Index.

The Conscript Register contains information about young men assessed for military service. Conscription includes physical examination, health assessment and tests of exercise capacity. The results from maximal exercise test on cycle ergometer were used as outcome in this study. All men conscripted for military service in 1993–2001 and born in Sweden 1973–1983 were eligible for the study, but subjects without records of exercise capacity or perinatal risk factors were excluded (Figure 1). During that time period, conscription was mandatory and enforced by law, but men with severe handicaps or congenital malformations generally received an exemption. Other reasons for not being conscripted were moving out of the country, death or conscription during another time period. The study period was chosen because of changes in conscription rules and the availability of perinatal data in the MBR.

The Population and Housing Census 1990 is a register to which adults in Sweden reported facts about education, profession, household, income and family structure in 1990. It was mandatory for all citizens 16 years and older. The response rate was 97.5% [29].

In The Multigeneration Register blood relationship of all Swedish citizens is registered. It was used to identify mothers and fathers of the conscripts.

### Outcome

Exercise capacity was obtained from the Conscript Register and defined as the maximal load (W_max_, expressed in Watt, W) that the conscript could manage on cycle ergometer. Only conscripts with a normal electrocardiography and without diseases or injuries that could influence the results were allowed to perform the test. The test was performed according to the conscription protocol with initial workload determined by weight and estimated physical fitness (125 W for weight 70 kg, presumed average fitness, all levels described in File S1). After 5 min cycling on submaximal load with a pulse between 120 and 170, the load was increased by 25 W every minute as long as the conscript managed. The conscript was instructed to perform his maximum capacity.

### Risk Factors, Confounders and Co-variates

Perinatal risk factors were obtained from the MBR. Gestational age (GA) in complete weeks was estimated from the date of the last menstrual period [30]. Subjects with records of GA <22 or >45 weeks were excluded. GA was categorized into 5 groups: extremely preterm (<28 weeks), very preterm (28–31 weeks), moderately preterm (32–36 weeks), term (37–41 weeks) and post term (≥42 weeks). Birth weight (BW) was measured in grams. Values <300 g and >7000 g were judged misclassified and excluded. Birth Weight Standard Deviation Score (BWSDS) was used as a measure for degree of large- and small-for-gestational age. BWSDS was calculated using a Swedish reference for normal fetal growth [31] and BWSDS was divided into five groups: <−2SD, −2SD to <−1SD, −1SD to <+1SD, +1SD to <+2 SD and ≥+2SD. For a baby born at 30 weeks GA a difference in BWSDS from −2SD to +2SD would mean a difference in birth weight from 1200 g to 2100 g. BWSDS exceeding ±6SD were excluded.

In addition, the following variables from the MBR were obtained: maternal age (<20, 21–25, 26–30, 31–35, 36–39, >40 years), maternal origin (born in Sweden, other Nordic countries, other European countries, Asia or other countries including North America, South America, Africa and Oceania), parity (primi- or multipara) and singleton or multiple birth.

Body Mass Index (BMI <18, 18–24.9, 25–29.9, 30–35, >35 kg/m^2^) and blood pressure (mmHg) - measured in the right arm after 5 to 10 minutes rest in the supine position - were collected from the conscript register.

A health assessment was performed by a physician at conscription. Based on physical examination, previous and current medical history, the conscripts were categorized in 12 health levels with A being the highest category. Level A represents fully healthy individuals without any minor health problem. Every lower level adds one or more health problem from mild allergies to asthma and severe diseases (described in detail in File S2).

Income of parents, including salary and income from finances, business and land or forest area, was retrieved from the Population and Housing Census 1990. Income was grouped in quartiles, the first quartile was considered as low, second and third as average and fourth quartile as high income. In this study, the highest income of mother or father was used.

The parental educational level of the conscript was reported in 8 levels and defined as the highest of mother or father. Socioeconomic index (SEI) categorizes people according to occupation. There are 15 index categories ranging from leading position, farmers and workers to non-occupied. Each index category has a dominance in relation to the others that is defined based on the expected impact on the child’s SEI [32]. In the study a subjects’ SEI was set as the most dominant SEI of the parents.

### Statistical Methods

All data were presented using descriptive statistics, i.e. number of subjects, mean and standard deviation for continuous variables, and frequency and relative frequency (percentage) for categorical variables. Data were analysed using General Linear Model, Analysis of Variance, ANOVA, with gestational age, BWSDS, number of births, maternal country of birth, maternal age, parity, parental education, socioeconomic index, parental income, BMI, blood pressure, health status and age at conscription as fixed factors in univariate models and multivariate models. Least square means was used to calculate the point estimates in the multivariate model and thereby control for co-variates and evaluate the independent contribution by each factor to the outcome.

All tests were two-sided and p<0.05 was regarded as statistical significant. Analyses were performed using the STATISTICA software, version 9.0, Statsoft Inc., Tulsa, US.

## Results

### Population Characteristics

During the 1993 to 2001 period 428,092 men conscripted for military service, of who 395,164 (median age 18, range 18–26 years) were born in Sweden 1973–1983, comprising 71% of all male births these years. Most of the men born these years and not included in the study were conscripted during another period of time. After exclusion of subjects with missing or misclassified data, the analysed cohort consisted of 218,820 men (Figure 1). A comparison between the characteristics of conscripts in the study cohort, who performed the maximal exercise capacity test and non-eligible subjects, is presented in Table 1, 2 and 3. The most striking finding was the significantly lower proportion with full health (A-status) among men who did not perform the exercise test (Table 3).

**Table 1:** Perinatal characteristics of analyzed cohort vs. non-eligible subjects.

	Analyzed cohort		Non-eligible	
	N = 218,820		N = 209,273	
	N	%	N	%
Gestational age (weeks)				
≤27	56	<0.1	109	<0.1
28–31	726	0.3	684	0.3
32–36	9,930	4.5	8,738	4.2
37–41	182,490	83.4	146,487	70.0
≥42	25,618	11.7	18,327	8.8
Missing	0	0.0	34,928	16.7
Birth Weight SDS				
<−2	7,052	3.2	5,418	2.6
−2 to <−1	35,747	16.3	27,536	13.1
−1 to <+1	147,819	67.6	117,797	56.3
+1 to <+2	22,970	10.5	18,473	8.8
≥+2	5,232	2.4	4,378	2.1
Missing	0	0	35,671	17.1
Multiple or single birth				
Singleton	215,055	98.3	173,026	82.7
Twins or N-tuplets	3,765	1.7	2,856	1.4
Missing	0	0	33,391	15.9
Maternal country of birth				
Sweden	201,160	91.9	190,843	91,2
Other Nordic countries	9,723	4.4	8,944	4.3
Europe	5,430	2.5	5,250	2.5
Asia	1,252	0.6	2,492	1.2
Other	820	0.4	1,344	0.6
Missing	435	0.2	400	0.2
Maternal age (years)				
≤19	11,749	5.4	8,678	4.1
20–24	62,074	28.4	47,169	22.5
25–29	84,041	38.4	64,287	30.7
30–34	46,104	21.1	40,591	19.4
35–39	12,955	5.9	13,195	6.3
≥40	1,897	0.9	1,962	0.9
Missing	0	0	33,391	16.0
Parity (number)				
1	92,902	42.5	71,302	34.1
≥2	125,918	57.5	104,580	50.0
Missing	0	0	33,391	16.0

**Table 2:** Parental characteristics of analyzed cohort vs. non-eligible subjects.

		Analyzed cohort		Non-eligible	
		N = 218,820		N = 209,273	
		N	%	N	%
Parental education					
Post-graduate		2,838	1.3	2,506	1.2
Post-secondary ≥3 years		41,479	19.0	34,392	16.4
Post-secondary <3 years		36,947	16.9	30,525	14.6
Upper secondary 3 years		31,989	14.6	26,625	12.7
Upper secondary ≤2 years		59,986	27.4	58,421	27.9
Primary/secondary 9–10 years		24,511	11.2	24,475	11.7
Primary/secondary <9 years		12,247	5.6	11,424	5.5
Unspecified		8,823	4.0	20,900	10.0
Missing		0	0	5	<0.1
Socio Economic Index (SIE)					
Self-employed professionals		10,111	4.6	7,146	3.4
Professionals/higher non-manual employees		35,948	16.4	28,910	13.8
Entrepreneurs		16,871	7.7	14,848	7.1
Intermediate non-manual employees		52,830	24.1	40,977	19.6
Assistant non-manual employees, lower level		19,788	9.0	16,103	7.7
Assistant non-manual employees, higher level		6,438	2.9	5,646	2.7
Skilled employees in goods production		27,978	12.8	26,750	12.8
Skilled employees in service production		7,675	3.5	7,668	3.7
Unskilled employees in goods production		12,032	5.5	13,557	6.5
Unskilled employees in service production		15,020	6.9	16,939	8.1
Farmers		5,638	2.6	4,062	1.9
Non-classified employees		4,155	1.9	5,565	2.7
Non-active gainfully employed		2,865	1.3	7,522	3.6
Missing		1,471	0.7	13,580	6.5
Parental Income					
High		96,400	44.1	72,258	34.5
Medium		104,237	47.6	97,511	46.6
Low		10,959	5.0	18,054	8.6
Missing		7,224	3.3	21,450	10.2

**Table 3:** Characteristics at conscription - analyzed cohort vs. non-eligible subjects.

	Analyzed cohort		Non-eligible	
	N = 218,820		N = 209,273	
	N	%	N	%
Body Mass Index (BMI intervals)				
<18	6,400	2.9	7,275	3.5
18–24.9	180,691	82.6	125,242	59.9
25–29.9	26,824	12.3	24,461	11.7
30–34.9	4,225	1.9	6,472	3.1
≥35	680	0.3	2,236	1.0
Missing	0	0	43,587	20.8
Systolic Blood Pressure (mmHg)				
–99	466	0.2	431	0.2
100–119	37,070	16.9	23,809	11.4
120–139	136,096	62.2	79,560	38.0
140–159	44,148	20.2	25,094	12.0
160–179	960	0.4	885	0.4
180–	80	<0.1	82	<0.1
Missing	0	0	79,412	37.9
Health Status				
A	128,081	58.5	45,997	22.0
B	14,564	6.7	9,304	4.4
D	32,121	14.7	18,973	9.0
E	11,210	5.1	16,589	8.0
J	7,848	3.6	15,830	7.6
JC	98	<0.1	5,498	2.6
Y	20,921	9.6	76,664	36.6
Z	750	0.3	3,139	1.5
Missing	3227	1.5	17,281	8.3

### Determinants of Exercise Capacity

The overall mean exercise capacity in young Swedish men was 306 W +/−50 SD. In the univariate analysis (ANOVA) there was a linear increase in exercise capacity with increasing GA (from 278 W in men born extremely preterm to 307 W in men born at term, p<0.001, Table 4). Exercise capacity also increased with increasing BWSDS (from 287 W in men with BW <−2 SD to 322 W in men with BW ≥+2 SD, p<0.001, Table 2A). Other factors affecting the outcome of maximal exercise capacity in univariate analyses were maternal country of birth, maternal age, multiple births, parental education, socioeconomic index and income of the parents, as well as the conscripts current BMI and systolic blood pressure (Table 4, 5, 6 and 7). All results in the univariate analysis were statistically significant, p<0.001.

**Table 4:** Maximal exercise capacity by perinatal characteristics (univariate analysis).

			Wmax (Watt)		
		N	Mean	SD	P
Multiple births					<0.001
Singleton		215,055	306	50	
Twins or N-tuplets		3,765	305	50	
Gestational age (weeks)					<0.001
≤27		56	278	55	
28–31		726	292	47	
32–36		9,930	302	49	
37–41		182,490	307	50	
≥42		25,618	306	51	
Birth Weight SDS					<0,001
<−2		7,052	287	49	
−2 to −1		35,747	296	49	
−1 to 1		147,819	308	49	
1 to 2		22,970	318	51	
>2		5,232	322	52	

SDS = Standard Deviation Score.

**Table 5:** Maximal exercise capacity by maternal characteristics (univariate analysis).

		Wmax (Watt)		
	N	Mean	SD	P
Maternal country of birth				<0.001
Sweden	201,160	307	50	
Other Nordic countries	9,723	301	49	
Europe	5,430	301	51	
Asia	1,252	288	48	
Unknown	435	294	52	
Other	820	299	49	
Maternal Age (years)				<0.001
–19	11,749	296	51	
20–24	62,074	303	50	
25–29	84,041	309	50	
30–34	46,104	309	50	
35–39	12,955	307	50	
40–	1,897	304	49	
Parity (number)				<0.001
1	92,902	307	50	
2+	125,918	306	50	

**Table 6:** Maximal exercise capacity by parental characteristics (univariate analysis).

		Wmax (Watt)		
	N	Mean	SD	P
Parental education				<0.001
Post-graduate	2,838	316	48	
Post-secondary ≥3 years	41,479	316	48	
Post-secondary <3 years	36,947	314	49	
Upper secondary 3 years	31,989	307	49	
Upper secondary ≤2 years	59,986	302	50	
Primary/secondary 9–10 years	24,511	298	50	
Primary/secondary <9 years	12,247	292	52	
Unspecified	8,823	298	51	
Socio Economic Index (SIE)				<0.001
Self-employed professionals	10,111	315	49	
Professionals/higher non-manual employees	35,948	315	48	
Entrepreneurs	16,871	304	50	
Intermediate non-manual employees	52,830	312	49	
Assistant non-manual employees, lower level	6,438	301	49	
Assistant non-manual employees, higher level	19,788	307	50	
Skilled employees in goods production	27,978	300	51	
Skilled employees in service production	7,675	303	49	
Unskilled employees in goods production	12,032	295	51	
Unskilled employees in service production	15,020	296	50	
Farmers	4,155	300	50	
Non-classified employees	5,638	304	49	
Non-active gainfully employed	2,865	289	51	
Missing data	1,471	299	47	
Parental income				<0.001
High	96,400	313	49	
Medium	104,237	302	50	
Low	10,959	296	50	
	7,224	298	51	

**Table 7:** Maximal exercise capacity by conscript characteristics (univariate analysis).

		Wmax (Watt)		
	N	Mean	SD	P
Body Mass Index (BMI intervals)				<0.001
−18	43,485	273	44	
18–25	143,606	313	47	
25–30	26,824	324	51	
30–35	4,225	314	51	
35–	680	307	58	
Systolic Blood Pressure (mmHg)				<0.001
–99	466	293	51	
100–119	37,070	297	52	
120–139	136,096	307	50	
140–159	44,148	313	48	
160–179	960	317	50	
180–	80	305	51	

**Figure 2:**
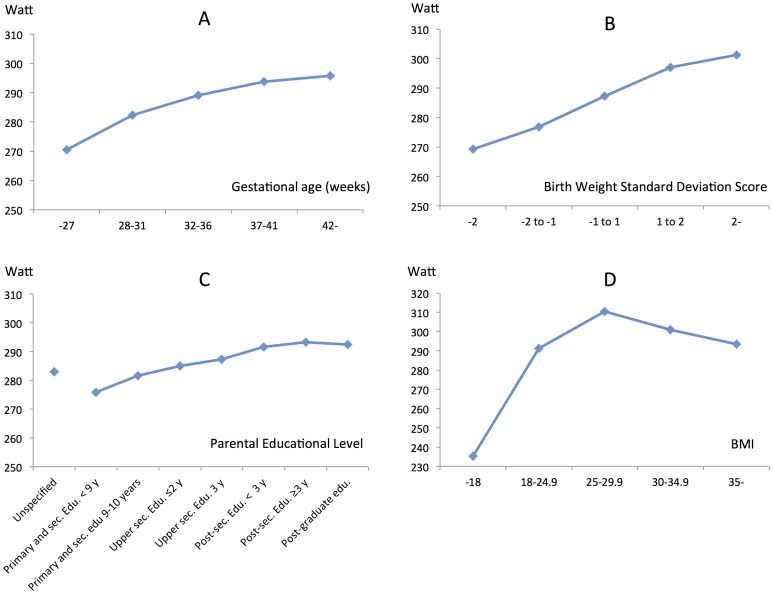
Maximal exercise capacity. Adjusted results expressed as least squared means for maximal exercise capacity (Watt) in relation to the major risk factors. A - Gestational Age categories, B - Birth Weight Standard Deviation Score, C – Education level of the parents and D - BMI. Sec edu. = Secondary education, y = years, BMI = Body Mass Index.

In the adjusted results from the multivariate analyses using general linear modelling, all levels of risk factors and categories of co-variates had significantly different effect on exercise capacity (p<0.001). The largest impact on W_max_ was found for GA, BWSDS, educational level of parents and current BMI (Table 8). Least square means for exercise capacity increased with increasing GA (≤27∶271 W, 28–31∶282 W, 32–36∶289 W, 37–41∶294 W, ≥42∶296 W, Table 8, Figure 2). Exercise capacity increased with BWSDS as follows: <−2SD: 269 W, −2 to −1∶277 W, −1 to +1∶287 W, +1 to +2∶297 W, >+2∶301 W (Table 8, Figure 2). The interactive effect of low gestational age and low birth weight for gestational age was tested and found to be low. Maximal exercise capacity increased with higher parental educational level, p<0.001 (Table 8, Figure 2). There was an association between BMI at conscription and the results on cycle ergometer (Table 8). The highest exercise capacity was achieved by conscripts with a BMI 25–29.9 (LS mean 310 W) followed by the conscripts with a BMI 18–24.9 (LS mean 291 W). The lowest results were achieved by subjects being underweight, with a BMI≤18 (LS mean 235 W) (Table 8, Figure 2). The BMI, body weight and height at conscription according to gestational age categories are listed in File S3.

**Table 8:** Maximal exercise capacity and major risk factors (General Linear Modeling).

	LSMean	SE	95%LL	95%UL	N	P
Gestational age (weeks)						<0.001
≤27	271	6	258	283	56	
28–31	282	2	278	287	726	
32–36	289	1	287	292	9,93	
37–41	294	1	291	296	182,49	
≥42	296	1	293	298	25,618	
Birth Weight SDS						<0.001
<−2	269	2	266	273	7,052	
−2 to −1	277	2	273	280	35,747	
−1 to 1	287	2	284	291	147,819	
1 to 2	297	2	293	301	22,970	
>2	301	2	297	305	5,232	
Parental education						<0.001
Post-graduate	292	2	288	296	2,838	
Post-secondary ≥3 years	293	2	290	297	41,479	
Post-secondary <3 years	292	2	288	295	36,947	
Upper secondary 3 years	287	2	284	291	31,989	
Upper secondary ≤2 years	285	2	281	289	59,986	
Primary/secondary 9–10 years	282	2	278	285	24,511	
Primary/secondary <9 years	276	2	272	280	12,247	
Unspecified	283	2	279	287	8,823	
Body Mass Index (BMI intervals)						<0.001
<18	235	2	232	239	6,400	
18–25	291	2	288	295	180,691	
25–30	310	2	307	314	26,824	
30–35	301	2	297	305	4,225	
>35	294	3	289	298	680	

Adjusted results expressed as Least Square Mean (LS Mean). SDS = Standard Deviation Score.

## Discussion

This is the first study to report that preterm birth predicts reduced exercise capacity in young healthy men, also after accounting for fetal growth. The association to preterm birth was found to be independent of other determinants of adult exercise capacity such as parental education, socioeconomic status and income, as well as of current BMI. The association was not limited to those men born very or extremely preterm, instead exercise capacity increased linearly with GA in a dose-response related manner. The effect size of being born preterm equalled or exceeded that of other known risk factors for unfitness in young adults, such as low parental education [33] and overweight [14].

The strengths of this study were the population-based design and the long-term follow-up, where prospectively collected data at birth could be linked to outcome 18–25 years later. The study was also large enough to assess inter-relationships between perinatal and other major determinants (parental education, socioeconomic status, income and current BMI) of exercise capacity in adulthood, while adjusting for maternal origin and multiple births. Importantly, we had sufficient power to separate the effects of preterm birth and fetal growth restriction on later exercise capacity. Low BWSDS - a proxy for poor fetal growth - was also found to predict low exercise capacity in young men. Accordingly, preterm birth is a perinatal risk factor for low exercise capacity in males later in life, even when taking fetal growth into account.

We have previously found that the conscription rate varied across the GA range, with the lowest conscription rate among men born extremely preterm [7]. Moreover, not all men who underwent conscription performed the exercise test. Comparing men who performed the exercise test with those who did not, the most striking finding was the significantly lower proportion with full health (A-status) among men who did not perform the exercise test (Table 3). This means that the conscripts who performed the maximal exercise test was a selection of the healthiest, which may in fact lead to an underestimation of the effect of preterm birth on adult exercise capacity in young men, particularly if applied to a more contemporary and unselected cohort of preterm survivors. Neonatal survival after preterm birth in 1973–1983 was lower than today and the neonatal period following prematurity was likely to be more traumatic. Hence, the effect of preterm birth on long-term fitness could have been expected to be greater than what we found in the present study. In view of the improvement of medical treatment in the neonatal period over time, the question has to be raised if the results of the present study are applicable on individuals born preterm in more recent years. Several circumstances indicate that the results can be expected to hold true. First, the survivors of prematurity in this study are likely to have been the healthiest, comparable to those with an uncomplicated neonatal course today. Second, exercise capacity is lower also in subjects born moderately preterm, a group less likely to require full intensive care. Third, there are several underlying physiological mechanisms irrespective of medical treatment that may explain a lower level of physical fitness in subjects born preterm [34]–[38]. As conscription for military service is no longer compulsory in Sweden after 2001, it is not possible to replicate this study in subjects born after 1983.

Yet another aspect that may affect the outcome on exercise capacity in subjects born preterm is psychological function. Subjects born preterm have been shown to have psychological difficulties to a greater extent than term subjects [39], but the effects of difficulties during up-bringing, neurodevelopmental and social aspects certainly warrants further studies. The gender aspect is the most obvious limitation of this study and by the design the conclusions can only with certainty be applied to males. We can only speculate about the result in females. Some studies have shown lower morbidity after preterm birth in females than in males [40], suggesting that if exercise capacity was tested in a population of both males and females the difference between those born term and preterm would be less pronounced. Other important limitations of this study include the lack of data on neonatal morbidity, infant nutrition, postnatal growth and smoking habits (of mothers and conscripts), which all could have had an impact on health outcomes after preterm birth. Given that smoking is more common in families with low education and socioeconomic index [41], which were included in our models, some of the effect of smoking on exercise capacity is corrected for in our model.

Our data demonstrate a linear relationship between preterm birth and exercise capacity, suggesting but not proving causality. Underlying mechanisms could be that preterm birth stops normal development and triggers adaptations in tissues and organs, ultimately resulting in lasting physiological alterations such as fewer alveoli [34], lower capillary density [35] and smaller vascular tree [36] - all impairing maximal oxygen uptake and transport - as well as lower leg power and coordination problems [19]. Cardiac function and performance could be involved, as indicated by remodeling of the heart in lambs and young adults born preterm [37]. Furthermore, a common genetic explanation for both preterm birth and low exercise capacity cannot be excluded. Finally, variations in behavior and motivation may clearly play a role for the result of the cycle ergometer test [20].

In this study the difference in maximal work load between male subjects born at term (306 W) and those born extremely preterm (278 W) is comparable to the difference in maximal aerobic capacity for an average man and an average woman [42]. The size of the difference would be clearly notable if two subjects were performing simultaneously. PA is often associated with some kind of competition. One could speculate that a child or adolescent not being able to perform at the same level as his peers may choose other activities, leading to a vicious circle of lower PA and lower exercise capacity. The level of fitness or PA has been shown to affect all-cause mortality [14], [43], risk of diabetes [16] and obesity [18], indicating that the lower level of fitness in young adults born preterm may be a link in the causal chain against morbidity in later life. Interestingly, higher systolic blood pressure in our cohort of young healthy males did not appear to be associated with reduced exercise capacity – a finding that may need to be explored further.

Preterm birth is a public health problem affecting between 6–12% [44] of all pregnant women. So far, the rates of this pregnancy complication have not declined, if anything, the opposite has been observed. Advances in perinatal medicine have, however, contributed to high survival rates after preterm birth today and have also significantly lowered the gestational age of viability [2]. The concern is now that increased survival may come at the cost of later morbidity. Young Swedish men and women born preterm have recently been shown to suffer from an increased mortality from cardiovascular and pulmonary causes [12]. The underlying mechanisms are poorly understood but in this context, our findings add to decrease this gap in knowledge. Preterm birth is associated both with low exercise capacity and high blood pressure [7], [8] and glucose intolerance [9], [10], risk factors for diabetes and cardiovascular diseases. The lower exercise capacity may be among the contributing factors for persons born preterm to develop these diseases.

Being born small was also associated with lower exercise capacity in later life. This finding was independent of gestational age and suggests that the effect of intrauterine growth restriction on future exercise capacity is similar after term as well as preterm birth. It also underlines the fact that to avoid significant health problems in coming generations [45], providing adequate care of the pregnant woman, including maternal nutrition during pregnancy, should be given highest priority in settings with on-going under- and/or malnutrition.

In conclusion, at an age where physical fitness and capacity peaks, healthy men born preterm have a lower exercise capacity compared to men born at term, irrespective of socioeconomic factors and current BMI. Reduced exercise capacity may add to future health problems faced by children growing up today following preterm birth and may play a role in the causal chain towards overweight, diabetes and hypertension. Early intervention programs to improve physical fitness in children and adults born preterm may be of value to reduce the risks for later disease and improve life-long health. Further studies on this issue, as well as on the underlying mechanisms, are warranted.

### Ethical Approval

The study was approved by the Regional Ethical Review Board in Stockholm.

## Supporting Information

File S1 Instructions for cycle ergometer exercise capacity test.
(DOCX)

File S2 Explanation of codes classifying health status at recruitment.
(DOCX)

File S3 BMI, body weight and height at conscription according to gestational age.
(DOCX)
