# Supplementary material for: Long-Term Impact of Preterm Birth on Exercise Capacity in Healthy Young Men: A National Population-Based Cohort Study
Source: PLoS One. 2013 Dec 6;8(12):e80869. doi: 10.1371/journal.pone.0080869 (PMC3855651; doi:10.1371/journal.pone.0080869)
Supplement: File S1 — Instructions for cycle ergometer exercise capacity test. (DOCX) [file pone.0080869.s001.docx]

**Supplement 1. Exercise capacity test**

Initial load on cycle ergometer according to weight in kg:

Weight (kg) Watt

-52.9 75

53.0-64.9 100

65.0-76.9 125

77.0-89.9 150

90.0- 175

For subjects performing physical training on a regular basis, initial load is increased by 25 W. Overweight subjects will be allowed a 25 w reduction in initial load.
