# Supplementary material for: Long-Term Impact of Preterm Birth on Exercise Capacity in Healthy Young Men: A National Population-Based Cohort Study
Source: PLoS One. 2013 Dec 6;8(12):e80869. doi: 10.1371/journal.pone.0080869 (PMC3855651; doi:10.1371/journal.pone.0080869)
Supplement: File S2 — Explanation of codes classifying health status at recruitment. (DOCX) [file pone.0080869.s002.docx]

**Supplement 2. Explanation of codes classifying health status at recruitment.**

| Category of health problem | Health Status Category  (eg. Availability for duty due to health) | | | | | | | |
| --- | --- | --- | --- | --- | --- | --- | --- | --- |
|  | **A** | **B** | **D** | **E** | **J** | **JC** | **Y** | **Z** |
| Certain infectious and parasitic diseases | 8 | 8 | 7 | 6 | 5 | 5 | 2 | 0 |
| Neoplasms | 8 | 8 | 7 | 6 | 5 | 5 | 2 | 0 |
| Diseases of the blood and blood-forming organs and certain disorders involving the immune mechanism | 8 | 7 | 7 | 6 | 5 | 5 | 2 | 0 |
| Endocrine, nutritional and metabolic diseases | 8 | 7 | 7 | 6 | 5 | 5 | 2 | 0 |
| Mental and behavioural disorders | 8 | 7 | 7 | 6 | 5 | 5 | 2 | 0 |
| Diseases of the nervous system | 8 | 8 | 7 | 6 | 5 | 5 | 2 | 0 |
| Diseases of the eye and adnexa | 8 | 8 | 7 | 6 | 5 | 5 | 2 | 0 |
| Diseases of the ear and mastoid process | 8 | 8 | 7 | 6 | 5 | 5 | 2 | 0 |
| Diseases of the circulatory system | 8 | 8 | 7 | 6 | 5 | 5 | 2 | 0 |
| Diseases of the respiratory system | 8 | 7 | 7 | 6 | 5 | 5 | 2 | 0 |
| Diseases of the digestive System | 8 | 7 | 7 | 6 | 5 | 5 | 2 | 0 |
| Diseases of the skin and subcutaneous tissue | 8 | 7 | 7 | 6 | 5 | 5 | 2 | 0 |
| Diseases of the musculoskeletal system and connective tissue | 8 | 8 | 7 | 6 | 5 | 5 | 2 | 0 |
| Diseases of the genitourinary system | 8 | 7 | 7 | 6 | 5 | 5 | 2 | 0 |
| Pregnancy , childbirth and the puerperium | 8 | 7 | 7 | 6 | 5 | 5 | 2 | 0 |
| Certain conditions originating in the perinatal period | - | - | - | - | - | - | - | - |
| Congenital malformations, deformations and chromosomal abnormalities | 8 | 8 | 7 | 6 | 5 | 5 | 2 | 0 |
| Symptoms, signs and abnormal clinical and laboratory findings, not elsewhere classified | 8 | 7 | 7 | 6 | 5 | 5 | 2 | 0 |
| Injury, poisoning and certain other consequences of external causes | 8 | 8 | 7 | 6 | 5 | 5 | 2 | 0 |
| External causes of morbidity and mortality | 8 | 8 | 7 | 6 | 5 | 5 | 2 | 0 |
| Factors influencing health status and contact with health services | 8 | 8 | 7 | 6 | 5 | 5 | 2 | 0 |
| Psychological Assessment at enrolment | 8 | 8 | 7 | 6 | 5 | 5 | 2 | 0 |

Grading:

8 = No functional reduction, Insignificant disorder 1 = Functional Reduction not defined

7 = Mild functional Reduction 2 = Severe functional Reduction

6 = Intermediate functional Reduction 0 = Extreme functional Reduction

5 = Considerable functional Reduction
