# Supplementary material for: Long-Term Impact of Preterm Birth on Exercise Capacity in Healthy Young Men: A National Population-Based Cohort Study
Source: PLoS One. 2013 Dec 6;8(12):e80869. doi: 10.1371/journal.pone.0080869 (PMC3855651; doi:10.1371/journal.pone.0080869)
Supplement: File S3 — BMI, body weight and height at conscription according to gestational age. (DOCX) [file pone.0080869.s003.docx]

**Supplement 3.**

**BMI, body weight and height at conscription according to gestational age**

| **Gestational age (weeks)** | **-27** | **28-31** | **32-36** | **37-41** | **42-** |
| --- | --- | --- | --- | --- | --- |
| **N** | 56 | 726 | 9,930 | 182,90 | 25,618 |
| **Weight (kg)** | 67 (10) | 69 (10) | 71 (11) | 72 (11) | 73 (11) |
| **Height (cm)** | 176 (6) | 177 (7) | 179 (7) | 180 (6) | 180 (6) |
| **BMI** | 22 (3) | 22 (3) | 22 (3) | 22 (3) | 22 (3) |

Expressed as mean (SD)
